# Between Rhetoric and Reality: Learnings From Youth Participation in the Adolescent and Youth Health Policy in South Africa

**DOI:** 10.34172/ijhpm.2022.6387

**Published:** 2022-04-25

**Authors:** Tanya Jacobs, Asha George

**Affiliations:** ^1^School of Public Health, University of the Western Cape, Cape Town, South Africa.; ^2^School of Public Health Faculty of Community and Health, University of the Western Cape, Cape Town, South Africa.

**Keywords:** Youth Participation, Health Policy, Policy Process, Actors, Youth, South Africa

## Abstract

**Background:** Youth participation makes an essential contribution to the design of policies and with the appropriate structures, and processes, meaningful engagement leads to healthier, more just, and equal societies. There is a substantial gap between rhetoric and reality in terms of youth participation and there is scant research about this gap, both globally and in South Africa. In this paper we examine youth participation in the Adolescent and Youth Health Policy (AYHP) formulation process to further understand how youth can be included in health policy-making.

**Methods:** A conceptual framework adapted from the literature encompassing Place, Purpose, People, Process and Partnerships guided the case study analysis of the AYHP. Qualitative data was collected via 30 in-depth, semi-structured interviews with policy actors from 2019-2021.

**Results:** Youth participation in the AYHP was a ‘first’ and unique component for health policy in South Africa. It took place in a fragmented policy landscape with multiple actors, where past and present social and structural determinants, as well as contemporary bureaucratic and donor politics, still shape both the health and participation of young people. Youth participation was enabled by leadership from certain government actors and involvement of key academics with a foundation in long standing youth research participatory programmes. However, challenges related to when, how and which youth were involved remained. Youth participation was not consistent throughout the health policy formulation process. This is related to broader contextual challenges including the lack of a representative and active youth citizenry, siloed health programmes and policy processes, segmented donor priorities, and the lack of institutional capability for multi-sectoral engagement required for youth health.

**Conclusion:** Youth participation in the AYHP was a step toward including youth in the development of health policy but more needs to be done to bridge the gap between rhetoric and reality.

## Background

**Key Messages**

** Implications for policy makers**

- Youth participation in policy-making is a right and involving young people in all that is relevant to them is part of global legal and policy commitment to end discrimination and exclusion and reduce inequalities.
- Policy-makers need to meaningfully engage youth in their diversity and in representative and accountable ways, in all stages and spaces of the policy-making process, as part of building youth citizenship and leadership.
- Understanding the dynamic relationships between context (place), people (actors) and processes, is crucial when analysing, planning and facilitating youth participation in policy processes.
- Multi-sectoral collaboration across government departments, with civil society, with researchers and with representative youth structures, can enhance the meaningful participation of young people in policy processes.
- A key area for action is for policy-makers to reimagine principles and ways of working, to ensure the enabling contexts, capabilities, resources, actors and processes are in place, to centre youth participation and bridge the gap between policy commitments on paper and lived realities of young people.

** Implications for the public**

Youth participation in policy-making is a right and it is very important to meaningfully involve young people in all policies that affect them, in order to build healthier, more just, and equal societies. It is really important to acknowledge the diverse experiences and voices of young people and include their participation in all stages and spaces of policy-making processes, beyond tokenism. A greater understanding of the benefits, *who* is involved and the *how* of ensuring participation in policy processes, will be good for youth, policy actors and all of society. As such, policy-makers should make sure that they create the enabling contexts, have the required capabilities, resources and partnerships, that put youth at the centre of all policies that affect them. We all need work together to ensure that young people, as current and future leaders, are not left behind in the Sustainable Development Goal (SDG) era.

The principle of ‘Leave no one behind’ is central to the 2030 Agenda for Sustainable Development and its Sustainable Development Goals (SDGs). Involving young people in all that is relevant to them is part of this global commitment to eradicate poverty in all its forms, end discrimination and exclusion, and reduce inequalities and vulnerabilities. Prior to the SDGs, youth participation was recognised as a right in global legal policy through the United Nations (UN) Convention on the Rights of the Child.^1^ This right underscores the importance of the involvement of children (defined as up to 18 years) in decisions, that affect them, including their health. The Convention on the Rights of the Child is often applied as the legal and policy foundation to encourage and legitimise youth participation as a civil, political, economic and cultural right and is complemented by the African Youth Charter as a regional commitment^2^ and other global policy frameworks such as, Beijing +25, and statements such as the Global Consensus Statement on Meaningful Adolescent & Youth Engagement.^3,4^ In South Africa, the National Development Plan and the new National Youth Policy (2020-2030) are aligned to the Constitution and to global and national rights policies which articulate youth participation as right.

 Participation is a right and it should be a priority to involve youth voices and policy beneficiaries as they can they make significant contributions and provide leadership in both programme and policy processes and meaningful engagement leads to healthier, more just, and equal societies.^3,5,6^ In addition to rights based legal and policy framework of youth participation as a right, there is increasing acknowledgement of youth participation as important for development of policies and programmes, including health.^5,7,8^ The meaningful engagement of young people in all aspects planning, implementing, monitoring and evaluating programmes and policies has multiple benefits for their own, and their communities’ health and development.^9-11^ From a health systems perspective, national policy frameworks need to recognize the meaningful engagement, participation and leadership of young people and understand them as active actors, not merely beneficiaries of health programming.^5,7^

 Despite the substantive global and national commitments to youth participation in policy processes on paper, several barriers and challenges exist in terms of youth participation in both health policy processes and programmes. Challenges include varied understandings and approaches related to young people, both in terms of diverse definitions and strategies to integrate them in decision-making, reflecting how constituencies and sectors shape adolescent policy priorities and programmes. Further, policy discourses about youth can be somewhat contradictory, constructing young people simultaneously as both ‘a risk’ to social cohesion and democracy and ‘a solution’ to ‘wicked problems.’^12^ Although various approaches have been used to engage and collaborate with youth in the development of health policies and programmes, significant gaps exist between policy-makers’ understandings of young people’s needs and their lived realities.

 In reviewing adolescent health policies in South Africa from 2003 to 2018,^13^ the content of the policy documents make reference to the South African Constitution and legal and policy frameworks that centre human rights, including commitments to health as a right, access to services, and addressing historical and current barriers to access for all South Africans. Participatory governance is an important right in the relatively recent democratization in South Africa.^14^ However, only three of the polices include explicit reference to the rights and empowerment of youth^15-17^ and only one mentions the engagement of youth in the policy development process, the Adolescent and Youth Health Policy (AYHP) (2017). The absence of youth participation in policy development processes across the health policy landscape in South Africa, is the backdrop and our paper provides contextual insights from the AYHP process to address this paucity. While the new National Youth Policy included consultation with youth across the nine provinces, scant details are provided on how this was done. Furthermore, delays in the process due to concerns raised by civil society actors, compounded by the coronavirus disease 2019 (COVID-19) pandemic, meant that the final policy was only released in March 2021.

 Definitions of youth participation and how terms such as “adolescents” and “youth” are applied are relevant starting points for understanding youth participation in policy processes. The UN defines youth participation as “the active and meaningful involvement of young people in all aspects of their own, and their communities’ development, including their empowerment to contribute to decisions about their personal, family, social, economic, and political development.” The World Health Organization (WHO) defines “adolescents” as 10-19 years and ‘‘youth’’ as those aged 15–24 years, and ‘‘young people’’ as being 10–24 years old.^7,18^ The African Youth Charter defines youth as 15-35 years and the South African National Youth Policy, defines youth as 14-35 years. Given the diverse terms and age ranges, in this this paper we refer to adolescent and youth, being from 10-24 years and use the term ‘youth’ to refer to range of adolescents and young people, as this is consistent with the age range referred to in the AYHP. Due to the inconsistent use of age ranges, proportions differ, but it is estimated that, the youth (15-34 years) constitute more than a third of the population. Adolescents (10-19 years) are estimated to make up 18.5% of the total population of South Africa.^19^

 There is a substantial gap between rhetoric and reality in terms of youth participation with scant research on youth participation in health policy-making, both globally and in South Africa. In this paper we describe and analyse youth participation in the AYHP process and also raise critical questions and lessons in terms of the *how* of youth participation in health policy-making. The key research question and lines of enquiry for this paper are: How was youth participation facilitated in the AYHP, which youth were involved and whose voices were heard, how was this facilitated, what was the context, who were the actors? What can be learned about strengthening youth participation in health policy overall? In addition, ‘zooming in’ on the AYHP and South African context is also a foundation to then ‘zoom out’ and engage with more meta questions in terms of youth participation in health policy-making processes more broadly, in order to identify learnings for those working in the fields of adolescent and youth studies, as well as health policy and systems research.

## Methods

 Several frameworks located in the intersecting fields of adolescent and youth health and critical youth studies explore youth participation in programmes.^5,12^ Over time the dominant frameworks on youth participation have included Arnstein’s “Ladder of Citizen participation,”^20^ Hart’s “Ladder of participation,”^21^ which has been adapted by several authors, including Treseder in 1997 and Shier.^22^ Wong et al developed the typology of youth participation and empowerment pyramid, where the ultimate aim was to achieve a balance between youth and adult control, through the empowerment of both, by establishing shared power relationships. The Lancet Commission framework for youth participation describe training and mentorship, adult partnerships, systems and resources as essential elements of meaningful youth engagement.^23^ These current frameworks are designed largely to monitor and evaluate youth participation in programmes, consequently there is an evidence gap in terms of youth participation in health policy processes.

###  Conceptual Framework 

**Figure 1:**
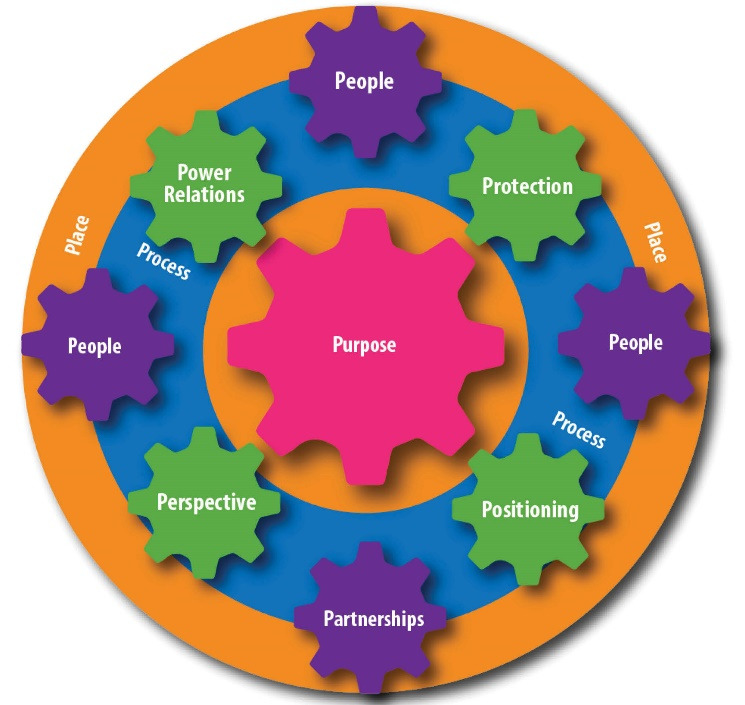
Conceptual Framework: Interactive and Dynamic Domains of Place, People, Purpose, Process, Positioning, Protection, Perspective, Power relations and Partnerships.

 Acknowledging and building on prior work conceptualising youth participation noted above, we expand and adapt a relatively new conceptual framework synergised from fields of feminist, post-structural and critical theory, as well as youth studies, and citizenship research for conceptualising and planning for youth participation in programmes.^12^ The model directs attention towards seven inter-connected domains of Purpose, Place, Process, Positioning, Protection, Perspective and Power relations. We expanded this model by adding 2 additional domains ie, P for People, the actors, as well as an additional P for Partnerships, across government departments and with civil society, for example. We also focus on Process, as a dynamic cross-cutting domain, and how it intersects with the domains of Positioning, Protection, Perspective, Power relations, Protection. The final conceptual framework (Figure 1) therefore has Place or context as the broader setting, which encompasses Process (Positioning, Perspective, Protection, Power relations) and Purpose, with People and Partnerships embedded throughout.

 Given the phenomenon of interest being youth participation in health policy processes, we therefore also integrate approaches from health policy analysis (HPA). Importantly, HPA goes beyond content and specifically considers the context that is shaped by individual, organisational, national and global factors, as well as the actors, in terms of their roles and influence on policy processes, at different levels.^24,25^ The Health Policy Triangle,^24^ which explores the dynamic interactions between content, actors, context and processes, was integrated into the interview guide. It has been used extensively at global and national levels and applied largely on public health concerns such as health human resources, services and systems, but not youth participation in policy processes.^26^ Mapping and understanding the actors, including youth, and how they interface with other actors, in health policy-making processes, is therefore an important line of enquiry, in order understand the complexity and dynamics of policy processes and to address the gap between rhetoric and reality.

###  Research Design 

 A case study design based on qualitative interviews was chosen which allowed for an enquiry of the phenomenon located in the social and political context, enabling sense-making of the complexity and nuances of context and processes that gave rise to and shaped youth participation, in the AYHP.^27,28^ These methods were used to unpack the research questions which were focused on describing the systems and processes of policy-making and exploring the perspectives of actors. Data was collected via in-depth, semi-structured interviews with a range of policy authors and actors. Through purposive sampling, AYHP policy authors in government, academia and donors, members of the Advisory Panel, youth representatives from the National Department of Health (NDoH) Adolescent and Youth Advisory Panel (AYAP), National Youth Development Agency, as well as youth health policy actors, in government, academia and civil society, representing a range of experiences and perspectives were identified (Table).

**Table:** Categories of Actors and Number of Participants Interviewed

**Category**	**Number **
AYHP author government	5
AYHP author academic	2
AYHP author international NGO funder	1
Government actor	6
AYHP Advisory panel members	5
Civil society actor	6
Academic actor	2
NDoH Adolescent and Youth Advisory Panel	3

Abbreviations: AYHP, Adolescent and Youth Health Policy; NGO, non-governmental organisation; NDoH, National Department of Health.

###  Data Collection

 Thirty respondents were interviewed between September 2019 and April 2021, in an iterative manner ie, after a first round of 15 interviews, initial analysis was conducted which guided subsequent interviews. The first round of interviews was conducted face-to-face, however in the COVID-19 context, the majority of the second round of interviews were conducted via the medium which participants preferred (Zoom, Google Meet, WhatsApp).

 Informed consent to interview and audio record was obtained from each participant and each interview was transcribed in full. Each participant was assured of anonymity and confidentiality.

 Reflexivity in understanding the researcher’s positionality in the research process is critical to understanding study design and findings.^29-31^ As part of our reflexive analysis, we were aware of how our power and positionality as two middle-class, South African academics, shaped the research process, including researcher-participant interactions and the power and privilege that is part of that positionality and is further reflected on in the discussion. For example, the lead author has twenty years of experience working in the field of adolescent health and therefore brought an understanding of the national context and histories that framed the topic. She also had relationships with many of the respondents that enabled access to key policy-makers and academics. Belonging to a university with a political history of advancing social justice, may have also influenced respondents willingness to participating in the research.

###  Data Analysis

 Data analysis was guided by the conceptual framework and the interview transcripts were analysed using thematic analysis, and included both deductive and inductive coding and categorisation.^32,33^ Initial deductive codes were based on Cahill and Dadvand^12^ mentioned above and included Purpose, Place, Process, Positioning, Protection, Perspective and Power relations. Through the thematic analysis, People and Partnerships were added as inductive codes, as they emerged from the data and the Conceptual framework was refined through this iterative process. In addition, interview data was triangulated both across respondents, as well as with data from document analysis of the AYHP undertaken for a previous paper.^13^

## Results

 The findings of youth participation in the AYHP are presented along the domains of Place, Purpose, People, Process, and how this included dynamic interactions between Positioning, Perspective, Protection, Power relations, as well as Partnerships, per the conceptual framework (Figure 1).

###  Place: ie, Context 

 The AYHP was developed in a national context where youth participation in policies relevant to their health had not taken place historically and there was therefore strong support for this as a ‘first’ and unique component, in the context of existing international and national commitments to the rights of young people, including the right to participate in policies and programmes that affect their lives.

 The AYHP timeline is contextualised in a history and current policy context that promotes proactive approaches to youth empowerment and health promotion, and was also intended to link the Integrated School Health Policy (2012) and the National Youth Policy (2015-2020). It is built on the foundations of earlier national health policies that focus on the health and wellbeing of adolescents and youth and this landscape includes the first National Policy Guidelines for Youth and Adolescent Health (2001), which was followed by the draft AYHP (2012). An internal NDoH review in 2014 highlighted critical gaps, such as lack of youth participation and evidence-based interventions. This was the impetus for the NDoH to initiate a new AYHP in 2015, in partnership with an appointed academic team to lead the technical support and with additional technical and financial support by the United Nations Population Fund (UNFPA) and was finalized in 2017.

 In addition, the AYHP is located in a policy context that is fragmented and uncoordinated, with several actors and unaligned policies relevant to adolescent and youth health and this was articulated by all types of actors. These co-exist and correspond to many vertical programmes both within the NDoH, within other government departments and with other national programmes. An example of this is the Adolescent and Youth Health Friendly Services (AYHFS), which was not really integrated into policy and programming: *“A review of AYHFS shows that it has not been successful after 20 years and the challenge remains in terms of where and how to create safe zones for adolescents within and beyond the health service and how services are not designed for young people”* (AYHP Author Government 14).

 The contextual realities of youth are shaped by key social and structural determinants, such as legacies of colonialism, apartheid, contemporary social and economic inequalities, as well as racial and gender inequality, which all intersect and compound each other. This context includes a historical denialism and neglect of the HIV epidemic, which resulted in delays and failures in the management of integrated HIV prevention, treatment and care, exacerbated by gender inequality, lack of access to education and training and unemployment. All the actors spoke to the history of intersecting social and structural determinants which construct the current realities and priorities of young people and also manifest in key challenges they face, as illustrated in the following quote, “*Young people experience intergenerational poverty and high levels of youth are not in educational employment or training. Gender-based violence [GBV] and the problem of violence, so those are the big things they face”* (AYHP Advisory Panel Member 11).

 With a post-Mbeki shift in political leadership that turned HIV policy around, the AYHP was developed in a context where HIV became a national priority, with a focus on adolescent girls and young women in particular, given the incidence data, with several corresponding government and donor initiatives addressing the interlinked national priorities of HIV and GBV. This is evidenced in the national She Conquers Campaign,^34^ the regional DREAMS (Determined, Resilient, Empowered, AIDS-free, Mentored and Safe) Initiatives, UN Agencies initiatives such as the She Decides campaign. All of these programmes and activities include a component of youth participation, often through ‘ambassadors’ and or ‘trail blazers’ and programmatic interventions, both with a focus on notions of ‘empowerment’ and agency, particularly young women, in the context of HIV and GBV epidemics.

 A key theme that emerged from many policy actors, is the concern that this type of youth participation has created a pattern and practice in South Africa, largely driven by HIV actors and donors. This includes positioning these individuals as social media influencers and ‘celebrities,’ that are not necessarily representative of diverse realities and youth civil society movements. Participants mentioned that while individual empowerment is important, it deflects from the substantive transformation of social and structural determinants and systems:“*They participate, they sit in forums and stand on platforms, so they make a very valuable contribution, but they are not necessarily connected back to a diverse youth constituency. They are basically eloquent people who happen to be young and there is a space for that, because I think obviously that is where the experts and the subject matter leaders of the future are going to emerged from. But those structures are often not connected downwards, in a way where there is accountability”* (AYHP Advisory Panel Member 21).

 Of further relevance is that, 27 years into South Africa’s constitutional democracy, the youth sector is still fragmented, includes some party political structures, but no organised, nationally representative civil youth structures and movements, as voiced by several policy actors from both civil society and government.

###  Purpose

 The purpose of youth participation was to identify and address adolescent and youth needs and priorities and was described in the AYHP document as central to the development. *“Youth participation and engagement have been central to this policy’s development” *(2017:2).Further, the purpose of participation, as a process of informing and gaining access to youth perspectives, was articulated by policy authors, “*It was very clear that we weren’t getting to what I thought were the key issues. So, we then switched gears and said, well, let’s ask young people”* (AYHP Author Government 14). Participation of youth in developing the AYHP was described as a unique, ground-breaking and ‘business unusual,’ across the spectrum of policy actors interviewed, including youth actors.

 Importantly, there are various nuanced actor perspectives in terms of the overall purpose of the policy and the role of youth participation in achieving this, as articulated by the participants. This continuum of perspectives includes the AYHP as a vehicle to operationalise the vision for youth health, meet the particular and changing needs of needs of youth, which had not been met by previous policies and services. In addition, another perspective includes that the overall purpose is to align policies, especially National Youth Policy and Integrated School Health Policy, as well as letting youth have a voice and involvement in the design and review of programmes.

###  People: ie, Actors 

 The actor landscape included the NDoH, as lead government actor and authors, who also triggered and managed the overall policy process. Additional lead authors included the academic team, as well as UNFPA. The academic team were selected based on their extensive experience in developing and facilitating participatory research with youth expert advisors. The authors were supported by an Advisory Panel comprised of key academics, researchers and other civil society actors in youth health, who led and contributed to the evidence reviews, as well as review of the AYHP. The absence of an AYHP policy champion to work across branches within the NDoH, as well as across departments and actors, in a structured mechanism and iterative manner, was highlighted by several policy actors.

 Youth, as key policy actors, participated in the policy process in particular ways and through existing research collaboration workshops and consultations, facilitated by the academic team (See Box 1). Youth included the Young Carers research groups, Mzantsi Wakho and the Sinovuyo Teen parenting programme. The NDoH also established an AYAP, consisting of one representative per province, that contributed to the policy development, as well as ongoing implementation advice and monitoring of services. Another layer of more distal actors includes key government departments, ie, Departments of Social Development and Basic Education, who participated in some of the AYHP consultative meetings. These actors also have an adolescent and youth health mandate, as well their own related policies and youth structures, that they consult and collaborate with.

**Box 1. Process of How of Youth Were Consulted as Part of the AYHP Development**

- Convening a Youth Health Parliament
- Visual exercises including ‘dream consultations’ and ‘dream clinics’
- Participatory research to investigate substance abuse, mental health/illness and adherence to chronic medicines
- Health clinic report cards in which adolescents and youth evaluated public health services
- Focus groups on sexual and reproductive health, intimacy, romance, risk and aspiration among youth and adolescents and their caregivers

--------------

Abbreviation: AYHP, Adolescent and Youth Health Policy.

Actors in government that did not participate in the AHYP include Departments of Higher Education and Training, Women, Youth and Persons with Disability, as well as key government led agencies, such as the South African National AIDS Council and the National Youth Development Agency. The South African context has multiple civil society actors of both youth-led and youth-focused groups and organisations, addressing several interrelated priorities such as youth development, HIV, Sexual Reproductive Health and Rights, and GBV, funded largely by donors and only a few were included in the stakeholder consultative processes. Importantly, less ‘visible,’ but powerful actors, including the funders such as Global Fund, ‘President’s Emergency Plan for AIDS Relief’ and the United States Agency for International Development, shape the programmatic agenda in South Africa, but were not directly included in the development of the AYHP.

###  Process, Including Positioning, Protection, Perspective and Power Relations 

 The process of making the policy included dynamic interactions between the domains of process, positioning, protection, perspective and power relations as per the conceptual framework. We describe what worked well, as well as what the challenges were in the sub-sections below.

####  What Worked Well 

 This sub-section describes what worked well during the process and in summary these include, there being a policy window, participation through established participatory research programmes, and building of relationships between policy actors. These are expounded upon in further detail below.

**Figure 2:**
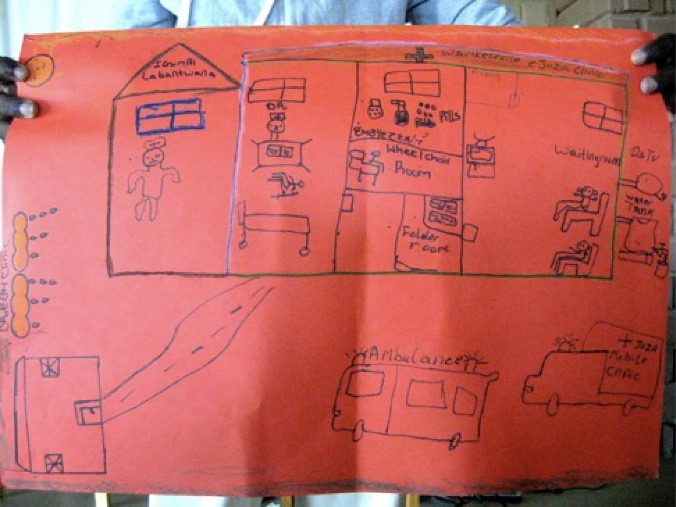
Example of a ‘Dream Clinic’. Source: AYHP (2017).

 A key enabling trigger was the policy window with government explicitly articulating and valuing involvement of youth and alignment with academic actors. Therefore, the positioning of young people was in terms of long standing participatory research programmes, being the Young Carers, Sinovuyo Teen research partnerships and the Mzantsi Wakho research programme, based in the Amathole District in the Eastern Cape.^35-38^ This positioning of youth as research participants in established participatory research programmes, had allowed relationships of trust to be built over time and their perspectives were gathered to establish the core objectives for the policy and the sixth objective makes direct reference to participation of youth to engage with policy and programming. See Figure 2 as an example of a Dream Clinic which included an ambulance, a mobile clinic, a good road, wheelchair room, a water tank and a comfortable waiting room.

 Further, NDoH senior staff also participated in some of these activities and this enabled building of relationships between academics, government and youth participants. This combination of a participatory research process, as well as generating evidence through reviews, was appreciated by the NDoH, and is illustrated by the quote: *“They loved that we had an empirical aspect of Mzanzi Wakho. So, we were going to be able to give them very strongly evidence-based data. But they also wanted something which would clearly have youth input*” (AYHP Author Academic 9).

**Figure 3:**
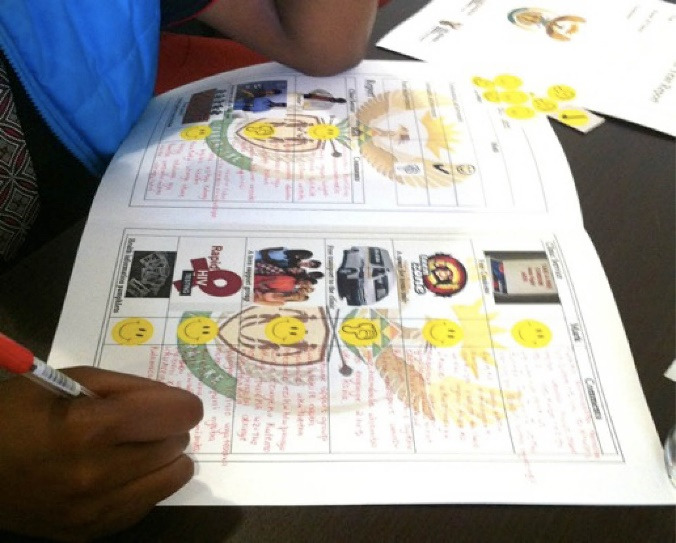
Health Clinic Report Card in Terms of for Example Staff, Services and Availability of Treatment.Source: AYHP (2017).

 In terms of power relations, the academic authors described the process of participatory research as being mindful of the voice and agency of young people and this is expressed in the quote, “*It was participatory and democratic and was following a kind of ethos of human rights and of engaging with children as powerful agents, not just subjects*” (AYHP Author Academic 9*).* An example of this was engaging youth in assessing clinics and giving them a report card using an inversion of authority as it is usually leaners who get report cards which were shared with the NDoH (See Figure 3).

 A further unique feature of the AYHP is that a team of academics were co-authors with the NDoH and there was a lot of leeway to develop the policy in an innovative way. This is expressed in the quote, “*The NDoH said specifically that the previous attempts had been too narrow and they wanted us to think outside the box, but didn’t really guide us on what that outside the box would be” (*AYHP Author Academic 10*). *

 Diverse perspectives exist in terms of the overall process, as many actors described the process as respectful and without obvious hierarchies and power relations, with well-intentioned lead authors. However, other actors’ perspectives included the lack of co-ordination and a national coordinating mechanism, poor stewardship, as well as it being an ad-hoc process, political fighting amongst actors, highlighting the complex and diverse perspectives as well as positionality of different actors.

####  What Were the Challenges 

 In addition to what worked well and the significant achievements, the AYHP process also raised certain key challenges in term of positioning and perspectives: how to embrace diversity and differences, how to manage power relations, as well as competencies and contexts for youth engagement. These are described in further detail below.

 Many policy actors raised critical questions about youth participation in the AYHP, that are also relevant to other policy processes in terms of the *how* of participation. Key questions highlighting important components of the planning, process and review of policy-making included: Which adolescents and youth were involved? Whose voices were heard? How was this facilitated? As stated, “*When thinking about policy-making processes we need to ask how participation was gained and how different constituencies of adolescents and youth were considered” *(AYHP Advisory Panel Member 11).

 In describing some of the challenges, policy actors noted that the youth that participated in the policy development process were not representative of youth diversity with different intersecting identities. For example, the need for inclusion of intersecting perspectives of youth across all geographical settings, LGBTIQA+ (lesbian, gay, bisexual, transgender, intersex, queer/questioning, asexual) young people, those living with disability, as well as across the age continuum, were voices and perspectives and representative structures, that were inadequately included in the AYHP process. Given the contextual landscape, several policy actors described that these diverse positionings and perspectives were not sufficiently taken into account during the process and that this also possibly constituted tokenism. A quote that captures this concern is *“Young people’s agency is now being expanded upon. Girl power, with all the different logos and buzz words. Young people are the future of the world and of our country and we need to provide them with the space and their participation in policy is laudable, but it’s also problematic because we have to recognise how age relations, gender, all impacts on who those voices are recognised” *(Academic Actor 27).

 In foregrounding power relations, some participants mentioned that in long term participatory research programmes, relationships of trust are built and this is conducive for youth to express themselves in supportive and facilitated spaces, also protected by research ethics. However, several actors noted the importance of always being aware of contextual power relationships between adults and young people, amongst young people, as well as the institutional power relations, when consulting with them in research, policy and programmes, as these are also shaped by the societal power relations.

 The need for consistent and sustained engagement of young people throughout the policy process was also highlighted by several actors, particularly in youth having decision making power in finalization of the AYHP. The following quote illustrates this, “So* in the decision-making processes towards, in the finalisation of the documents you found that there were technical experts from different universities and government departments and multilateral organisations, like the UNFPA. So, there were two very different processes. You had the consultations where they focused on getting the expertise of the youth demographic, but then when it comes to the decision-making and high level discussion platforms they were completely excluded, there needs to be consistency” (*NDoHAYAP Member 20).

 In addition to participation by youth as part of the process, there were broader process challenges related to who was ‘at the policy-making table,’ given time and resource limitations. As one of the authors noted, “*Lots of people weren’t involved and that was partly because we were just trying to get it done. There was quite a short deadline and we were just trying to get it done” (*Academic Author 10*).* However, other policy actors raised concerns about a lack of clarity and transparency in terms the inclusion of actors and stakeholders who were not consulted in the AYHP process. This was articulated follows, “*What I don’t know is how coherently all of that pulls together in terms of people really trying, how much do all the people who care about and would, could be positioned to influence policy or practice, are really sitting around a table?”* (Civil Society Actor 16).

 Further, the NDoH organisational context was described by several policy actors as being in ‘emergency mode’ and responding to health challenges, with little time to think, plan and reflect, including on youth participation in policy processes. Also, the importance of organisational processes, leadership and capacities for youth engagement, as well as collaboration with other sectors as routine, was raised as critical considerations, by several of policy actors. For example, this included the individual and organisational skills and competencies, commitment, alignment and systems to work with youth, within and across departments.

 Key reflections by actors during the interviews, on both the AYHP and other health policies, includes the importance of engagement with youth beyond health services and the health sector, meaningfully engaging diverse youth on topics such as changing social and economic and work realities, education systems, mental health, nutrition, climate change and gender equality, for example, to bridge the disconnect the between policy documents and lived realities to addressing some of the challenges in ensuring that there is an enabling environment. This is captured in the quote *“The first thing that we need to do, we need to stop thinking for young people. Through a lot of dialogues we need to find out what is it that young people want, not what we think that young people want” *(Government Actor 25). Digital modes were highlighted as potential ways to further engage youth, but critical concerns were raised of how this also potentially reinforces and exacerbates existing and intersecting inequalities, as highlighted in COVID-19 context.

 Several policy actors noted, that despite the commitment to ‘nothing about us without us,’ there in an evidence gap, as well as capacity deficit, in terms of *how* to engage youth in policy processes. This vexing question was articulated as follows, *“I am saying it is extremely complex and sometimes I think very often we try but I don’t think we succeed all the time. In fact, I think most times we don’t succeed and it would be really helpful to get some lessons and ideas and guidance of how we can do this better as policy-makers” *(Government Actor 17).

 In addition, several actors highlighted the importance of youth participation beyond tick-box exercises and once-off consultations, but that it requires enabling environments, financial and human resources and capacities of older and young people to be able to meaningfully, systematically and continuously, engage with young people from a rights framework. An illustrative quote is, “*I think the challenge, is that if you really want proper youth participation you need to have an enabling environment for them to participate and the tools, the support, the capacitation and the resources they need. So we can’t just come in and be part and parcel of a meeting or writing a policy document or having a once off consultation, but it needs to be an ongoing inclusion in that process” *(Government Actor 28).

###  Partnerships: Actor Interfaces and Multi-sectoral Collaboration 

 Youth Participation in the AYHP also foregrounded the sectoral contexts (eg, Health, Education, Social Development), actor interfaces (eg, between government departments and between government and civil society), as well as the importance of multi-sectoral partnerships, which are all relevant to how youth are engaged in policy and programmes.

 Within the NDoH, the health of youth is the mandate of several departments, including departments of adolescent and youth health and HIV. The HIV youth programme did not participate in the AYHP process and have their own youth consultation processes and structures, linked to Youth Councils at the South African National AIDS Council and through certain civil society actors that they fund.

 In addition to the internal consultation processes, the process of consulting with other government departments and actors was described as a challenging journey, as captured in the quote, “*Sometimes it was like climbing Mount Kilimanjaro, sometimes it was really difficult but at the end we managed to work together on the AYHP” *(AYHPAuthor Government 6). A further key theme that was highlighted by several policy actors, is the history of silo approaches, turf issues and lack of synergy in ways of working between key government departments and across various policy processes, relevant to youth. The following quote illustrates this: “*Government departments are weird things; people will not come and publicly announce that, yes thank you very much, if it was not for this group of people we would not have been able to do our job. They present it at the end as if they did it on their own”* (Government Actor 22).

 All policy actors raised concern about the challenges of collaboration and coordination across departments and the importance of a shared vision and this concern was expressed as follows, “*You can set up the processes but it needs the right leadership, the mindset of people, to be about working together for a common thing. The challenge is how to get people to work in teams” *(Government Actor 19*). *

 Importantly, in addition to the above, in the South African context there is a plethora of youth groups including youth-led and youth-focused, linked to government departments, donors, non-governmental organisations and civil society organisations, (eg, Siyakwazi Youth Network, Mmhoho campaign, Soul Buddyz Clubs, Agape youth movement, Sexual and Reproductive Justice Coalition and several HIV focussed youth structures), which contributes to inadequate coordination of youth-focused stakeholders. Given this range and multiplicity of actors, a key message from all policy actors interviewed, is the need for a dedicated, capacitated, national coordinating mechanism department, ideally led by the Presidency. In addition, several actors highlighted the current debates in terms of the underlying determinants of health and the roles of different sectors and this was captured as follows, “*Does the input need to be health for the output to be health? What we have learnt over the last decade is that often the input is something quite different or a combination of health and something quite different*” (AYHP Author Academic 10).The need for multi-sectoral co-ordination and collaboration was highlighted by all policy actors, particularly in how youth health is complex and by definition, requires the participation of multiple sectors and actors, including centering the meaningful engagement of youth themselves.

## Discussion

###  Summary of Main Findings

 This article examines the phenomenon of youth participation and the results draw attention to the complex nature of youth participation in the AYHP, which was a ‘first’ and unique component in health policy in South Africa.

 Despite various positive features, the experience also highlights various enduring challenges when facilitating youth participation in health policy-making. Youth participation was enabled by leadership from certain government actors and involvement of key academics with a foundation in long standing youth research participatory programmes. However, challenges related to when, how and which youth were involved remained. Youth participation was not consistent throughout the health policy formulation process. This is related to broader contextual challenges including the lack of a representative and active youth citizenry, siloed health programmes and policy processes, segmented donor priorities, and the lack of institutional capability for multi-sectoral engagement required for youth health. In addition, a key contextual factor is that some young people as treated as celebrity ambassadors, but without representation or accountability to the broader population of young people.

 Focussing on youth participation in the AYHP policy development process has also provided a concrete example for gaining insights into meta questions and lessons and these are further discussed below.

###  Participation as Right, Beyond Tokenism 

 The conceptual framework (Figure 1) and the results contribute to the intersections of youth studies and health policy literature, by deepening understanding of youth participation in policy processes and builds on existing scholarship.^5,12,39,40^

 A key message from our findings is the importance of moving beyond individual notions of youth participation and ‘celebrity’ status, to more systematic processes of routinely including the voices and agency of young people, in their full diversity, in all policies and programmes, which remains both an ambitious goal and a vexing challenge to implement in reality. An important theme that emerged from the findings is that of including perspectives of diverse young people, as an essential component of youth participation. This is crucial in a South Africa and global context where past and present social and structural determinants shape the health of young people and foregrounds challenging debates in the context of multiple actors, power relations and inequalities.

 Our findings are similar to that of Wigle et al,^10^ who describe the gaps of involving young people in SRH policy-making in Malawi and the importance of integrating youth in all stages of the policy-making, beyond tokenism, but as equal partners and experts on their health. This ‘first’ participation of youth in developing health policy in South Africa, highlights critical questions in terms of how to ensure youth participation, beyond being instrumental, but realizing the principles of meaningful engagement.^7,41^ The findings share some learnings of what worked well and what the challenges were and contributes to policy debates on understanding rights-based approaches to youth participation in all that affects them. This talks to the theme of youth participation as a right, as part of fostering citizenship and leadership, both in South Africa and globally.^42-47^

###  Implications for Policies, Programmes and Systems 

 The innovation of youth participation in health policy in South Africa is a step in the right direction, however our findings raise implications for how to include perspectives of diverse youth, meaningful participation in all stages of the policy-making processes, as well as required contextual and organisational systems, as also highlighted by other authors.^11,48^

 Linked to the point above of avoiding tokenism when youth participate in policy processes, the AYHP process also generates lessons and insights into participation of policy beneficiaries and how they can make contributions and leadership through their position and in realising the ‘Leave no one behind’ principle. This point is also made by Campbell et al,^11^ who discusses the importance of participation of networks of HIV positive youth in HIV programmes, as well as by Peta^49^ and Ngunyen et al,^50^ who describe how girls living with disabilities, can participate in policy processes.

 A policy window opened for youth participation through the AYHP, but it took place in a complex and dynamic context of multiple actors, with its own particular momentum, urgency and time pressures. The AYHP process highlights some of the tensions and complexities of managing policy development processes and the interactions between place, people and processes, how this shapes youth participation, in terms of who and what is included in the final policy, without organised youth health actors and youth citizenry.^51,52^ Therefore, the AYHP youth participation process also highlights how policies are socially constructed, the importance of good intentions and building of relationships between researchers and policy-makers, as well as the challenges in terms of balancing urgency versus more democratic and deeper processes, as part of the interplay between ideas, interest and institutional processes.^25,53^ Further, it raises critical questions in terms of the purpose of youth participation, that should be based on rights principles and can contribute to policy and programmes, but that consultation does not replace investments in enabling deeper engagement and other health systems challenges.

###  Process: Capacities, Organizational Architecture and Power Relations

 The results provide insights into the organisational architecture of youth participation and underscores the necessity to strengthen capacities, necessary platforms and the training, ongoing mentorship needed, as also highlighted by others.^12,54^ As policy-makers, researchers and young people, we need to prioritise the competency gaps and determinants of youth participation to ensure sustained, deep and meaningful ways, beyond just a few youth ambassadors and ‘older’ experts in policy processes.

 In addition to the enabling contexts and organisational architecture, our findings also reiterate the need for shifts in mindsets, paradigms, developing innovative partnerships and capacity strengthening for government and civil society, as well as resources, for ethical youth engagement in South Africa, as well as at the global level.^55,56^

 The results have implications for health systems and provides insights into how actors interface in relation to policy processes, and illustrates that the policy processes are also a function of power and politics at play. This includes, both ‘politics’ ie, micro politics in interpersonal relationships, different ideas and power relations between government actors, academic researchers, donors, young people and civil society, as well as between government departments, and how these interact with ‘Politics,’ ie, macro politics of the country and layers of social and historical contexts of South Africa.^57,58^ In addition, this research has described some of complexities of policy processes that happen and provide insights into the contours and dimension of agenda setting and the processes before implementation, as also analysed by other HPA scholars.^59,60^

 Further, our findings talk to themes of power and problematizes concepts such as ‘empowerment,’ ‘youth participation’ beyond the buzzwords and mantras, also re-framing young people from “passive” and “recipients” to “capable” and “active,” which opens up possibilities to re-imagine policy processes. It is aligned to what Gaventa and Cornwall^61-63^ have written in terms power relations and the spaces for participation. We would argue that there is need for further analysis of spaces and relationships for participation at micro, meso and macro levels and how these are embedded in broader unequal social systems.

 Importantly, the results open up significant debates on multi-sectoral collaboration, which is largely the terrain of governance, and essentially about brokering and sustaining these complex relationships and interactions, as described in the literature.^64-69^ Similarly, our results point to the importance of a shared vision, leadership, relevant capabilities and co-ordination across government and civil society actors, in working with youth, to ensure alignment of policies and programmes in the SDG era.

###  Positioning and Research Processes 

 To our knowledge, this research is the first to apply and adapt the Cahill and Dadvand^12^ framework, which could also be the basis for further empirical studies. By telling the story of youth participation in the AYHP and how this was shaped by domains of Place, People, Partnerships and Processes for example, opens up several research opportunities. A priority is research that is located in the synergies between youth studies and HPA, particularly, focussing on policy processes that facilitate meaningful participation, as well as actor and power analyses.^70-72^ There is a significant gap between what policy-makers think and the imaginations, experiences and realities of young people and research can contribute to addressing this.

 The AYHP has demonstrated that youth participation is possible through long standing research partnerships and is a foundation on which to build and contextualise youth as expert actors, as a more proactive approach to youth engagement in policy processes and programmes. As documented by Cluver et al^73^ participatory research approaches, such as youth participatory action research, photo-voice, and digital modes, can acknowledge and attempt to address the power imbalances that privilege researchers and adult perspectives and agendas, as well as challenge top-down policy development.^74-79^ However, it is also important to be aware of the relevant methodological, pedagogical approaches as well as tensions, power relations and potential resistance by policy-makers and other actors.^11,80–84^ In this way researchers can play a role as mediators, facilitators and partners, to address the research gaps, development of toolkits/resources and contribute evidence.^85^

 As part of our reflexive analysis throughout the research process, we were aware of how our positionality including having power as researchers and the privilege to undertake the study, provided the opportunity of speaking with a range of diverse youth health policy actors, who should ideally be in conversation with each other. As part applying the principles of reflexivity, we constantly reflected on how our Health Policy and Systems Research analytical lenses, power and privilege as academics shaped our relationship with the actors and the research process and results as a whole. While the extensive practitioner experience of the lead author brought deeper understanding of context to the issue under study, respondents may have also been more open to talking adolescent health issues knowing your background.

 Strengths of this paper include that it presents perspectives of a range of AYHP authors, as well as policy actors concerned with youth health, including the AYAP members involved in the policy process. However it also has limitations, in that it does not include perspectives of representative and diverse youth and structures in the general population, and this could be an area for future research. Also, youth from the Mzantsi Wakho and Sinovuyo Teen participatory research programmes were not interviewed, as their confidentiality is protected as part of the research ethics.

 Looking ahead, an essential element is a mobilised, capacitated, diverse youth citizenry as important actors to ensure youth participation, and the use of available tools and resources and guidance in a reflexive manner.^86-90^ Building on Cahill and Dadvand,^12^ we also suggest a list of prospective questions to guide youth participation in policy processes that can be used by a range of actors eg, policy-makers, youth focused/led organisations and researchers (Box 2).

**Box 2. Questions to Guide Youth Participation**

- *Place:* How will you consider and respond to the role of context and the social and structural determinants of both youth health and youth participation?
- *Purpose: *What contribution to policy development do you want to achieve through youth participation and how will you ensure that?
- *People:* Who are all the actors and stakeholders involved and how will you map, engage and manage them?
- *Positioning:* How will you get young people to participate? How will you consider their positioning within the wider context and in relation to others, within broader democratic processes?
- *Perspectives:* How will you embrace diversity and difference and how facilitate/ensure that perspective of diverse young people in terms of eg, age, location gender identity and sexual orientation, dis(ability) etc, are included?
- *Protection: *How will you ensure safety and ensure that their rights are respected, upheld and protected?
- *Power relations:* How will you build inclusion and respect and manage power relationships between actors in terms of interpersonal as well as within the institutional practices and structures?
- *Process:* What approaches, pedagogies and methods will you use and do you have the sufficient competencies and resources to enable a sustained process?
- *Partnerships:* What partnerships and institutional spaces and mechanisms exist and how will you manage these/do you have the competencies to manage these?

## Conclusion

 This paper sought to describe youth participation in the AYHP and draw out lessons to bridge the gap between rhetoric and reality, so doing responds to the call for support from policy-makers on the *how* of meaningful participation and leadership of young people in policies. Dynamic and complex relationships exist between place, people, partnerships and processes and this shaped how the AYHP was developed in South Africa, which was a novel and unique step toward including youth in development of health policy. Despite these achievements and steps in the right direction, several vexing questions and lessons were identified in terms of how to ensure meaningful participation of diverse young people across all stages of and spaces of policy-making. A key learning from this research is that policy-makers need to meaningfully engage youth in their diversity and in representative and accountable ways, in all stages and spaces of the policy-making process, as part of building youth citizenship and leadership.

 We add to the call for the reimagining of new paradigms, policy processes and systems which give more power to young people, as an important and powerful demographic. Leaving no one behind in the SDG era means ensuring the enabling contexts, resourced and sustained processes, appropriate competencies that centre leadership and voices of young people, in all policies and programmes that matter to them.

## Ethical issues

 This article is part of a larger PhD research case study titled: People, power and processes a gender analysis of adolescent health policy in South Africa which has received ethical approval by the Biomedical Science Research Ethics Committee of the University of the Western Cape (Reference number: BM18/9/9).

## Competing interests

 Authors declare that they have no competing interests.

## Authors’ contributions

 Both TJ and AG developed the conceptual framework and TJ collected the data. Both authors contributed to the analysis and interpretation of the findings and the drafting of the paper. Both authors read and approved the final manuscript.

## Funding

 This work was supported by funding from the South African Research Chair’s Initiative of the Department of Science and Technology and National Research Foundation of South Africa (Grant No 82769) and the South African Medical Research Council. Any opinion, finding and conclusion or recommendation expressed in this material is that of the author and the NRF does not accept any liability in this regard. This paper is part of the work undertaken by the Drivers Technical Working Group from Countdown 2030 funded by the Bill and Melinda Gates Foundation INV-007594/OPP1148933.
